# Targeting lysosomal cysteine protease cathepsin S reveals immunomodulatory therapeutic strategy for oxaliplatin-induced peripheral neuropathy

**DOI:** 10.7150/thno.54793

**Published:** 2021-03-04

**Authors:** Szu-Jung Chen, Li-Hsien Chen, Yu-Min Yeh, Chou-Ching K Lin, Peng-Chan Lin, Han-Wei Huang, Meng-Ru Shen, Bo-Wen Lin, Jeng-Chang Lee, Cheng-Che Lee, Yueh-Feng Lee, Huai-Chueh Chiang, Jang-Yang Chang

**Affiliations:** 1 Department of Internal Medicine, National Cheng Kung University Hospital, College of Medicine, National Cheng Kung University, Tainan, Taiwan.; 2 Department of Neurology, National Cheng Kung University Hospital, College of Medicine, National Cheng Kung University, Tainan, Taiwan.; 3 Department of Obstetrics/Gynecology, National Cheng Kung University Hospital, College of Medicine, National Cheng Kung University, Tainan, Taiwan.; 4 Department of Pharmacology, College of Medicine, National Cheng Kung University, Tainan. Taiwan.; 5 Department of Surgery, National Cheng Kung University Hospital, College of Medicine, National Cheng Kung University, Tainan, Taiwan.; 6 School of Medicine for International students, College of Medicine, I-SHOU University, Kaohsiung, Taiwan.; 7 National Institute of Cancer Research, National Health Research Institutes, Zhunan, Taiwan.; 8 Institute of Biotechnology and Pharmaceutical Research, National Health Research Institutes, Zhunan, Taiwan.

**Keywords:** Oxaliplatin-induced peripheral neuropathy, Cathepsin S, olfactory receptor transcription factor 1, IL-10

## Abstract

**Rationale:** Oxaliplatin-induced peripheral neuropathy (OIPN) is a common adverse effect that causes delayed treatment and poor prognosis among colorectal cancer (CRC) patients. However, its mechanism remains elusive, and no effective treatment is available.

**Methods:** We employed a prospective cohort study of adult patients with pathologically confirmed stage III CRC receiving adjuvant chemotherapy with an oxaliplatin-based regimen for investigating OIPN. To further validate the clinical manifestations and identify a potential therapeutic strategy, animal models, and *in vitro* studies on the mechanism of OIPN were applied.

**Results:** Our work found that (1) consistent with clinical findings, OIPN was observed in animal models. Targeting the enzymatic activity of cathepsin S (CTSS) by pharmacological blockade and gene deficiency strategy alleviates the manifestations of OIPN. (2) Oxaliplatin treatment increases CTSS expression by enhancing cytosol translocation of interferon response factor 1 (IRF1), which then facilitates STIM-dependent store-operated Ca^2+^ entry homeostasis. (3) The cytokine array demonstrated an increase in anti-inflammatory cytokines and suppression of proinflammatory cytokines in mice treated with RJW-58. (4) Mechanistically, inhibiting CTSS facilitated olfactory receptors transcription factor 1 release from P300/CBP binding, which enhanced binding to the interleukin-10 (IL-10) promoter region, driving IL-10 downstream signaling pathway. (5) Serum CTSS expression is increased in CRC patients with oxaliplatin-induced neurotoxicity.

**Conclusions:** We highlighted the critical role of CTSS in OIPN, which provides a therapeutic strategy for the common adverse side effects of oxaliplatin.

## Introduction

Oxaliplatin has exhibited significant efficacy in the treatment of human cancers, especially colorectal cancer (CRC). However, acute or chronic peripheral neuropathy and concurrent sensory and motor function deficiencies are a common adverse effect of oxaliplatin 1, resulting in chemotherapy dose reduction and early discontinuation 2. Several mechanisms of oxaliplatin-induced peripheral neuropathy have been proposed, including direct damage, ROS generation, and dysfunction of voltage-gated ions channels in the dorsal root ganglia and axon 3, 4. Several clinical trials were conducted to prevent or treat oxaliplatin-induced peripheral neuropathy based on the aforementioned mechanisms. However, these studies failed to demonstrate significant effectiveness in alleviating this side effect 5, 6. Moreover, increasing evidence suggests that neuroinflammation also plays a role in the development of neurotoxicity 7. In animal studies, inhibiting macrophage infiltration and proinflammatory cytokines in the dorsal root ganglion (DRG) suppressed the augmentation of paclitaxel-induced neuropathic nociception 7, 8. Milligan *et al.* reported that intrathecal administration of anti-inflammatory cytokine Interleukin-10 (IL-10) briefly reversed chronic constriction injury-induced mechanical allodynia and thermal hyperalgesia 9, 10. Furthermore, suppression of IL-20, a proinflammatory cytokine, with IL-20 neutralizing antibody alleviated paclitaxel-induced peripheral neuropathy in animal models 11. Therefore, triggering anti-inflammatory cytokines and suppressing the proinflammatory response may be a therapeutic strategy for managing chemotherapy-induced peripheral neuropathy (CIPN).

Cathepsin S (CTSS), a lysosomal cysteine protease, is stably present in the cytoplasm of immune-related cells, such as antigen-presenting cells, B cells, and phagocytic cells (e.g., macrophages and microglial cells). Several lines of evidence have demonstrated that releasing CTSS from microglial cells causes neuropathic pain 12 that could be reversed by intraspinal injection of CTSS inhibitor, but the mechanisms remain unclear. Furthermore, CTSS is reportedly an autocrine factor that stimulates microglial cell activation and subsequently regulates the release of proinflammatory cytokines and chemokines 13. Our studies have indicated that CTSS plays a vital role in mediating Ca^2+^ homeostasis by disturbing stromal interaction molecule 1 (STIM1) trafficking, which alters STIM1-Orai1 interaction and affects Ca^2+^ influx 14. And, studies have reported that STIM1 regulates stored-operated Ca^2+^ entry (SOCE) by initiating Ca^2+^ influx and triggering immune activation in macrophages, chemotaxis, and neutrophil infiltration, which plays a role in the development of inflammatory pain 15.

IL-10, a “founding” member of IL-10 family cytokines, was initially described as a cytokine synthesis inhibitory factor produced by T helper cells with pleiotropic immunosuppressive functions 16. Increasing evidence indicates that IL-10 is expressed by a wide variety of cell types, including macrophages, monocytes, dendritic cells, natural killer cells, and CD4^+^ and CD8^+^ T cells and B cells. The immunosuppressive activity of IL-10 is mediated by ligand binding with IL-10 receptor (IL-10R1, IL-10R2), which activates Janus tyrosine kinases (JAKs) and signal transducers and activators of transcription signaling pathways, which inhibit the expression profile of immunomodulatory genes 17 and release proinflammatory cytokines, such as IL-6, IL-1β, and TNF-α 18. However, despite the fact that IL-10 acts as a prime drug target for autoimmune disease, tissue damage, and even cancer, effective therapies have yet to be developed.

Therefore, we investigated whether targeting CTSS can ameliorate oxaliplatin-induced peripheral neuropathy (OIPN) by upregulating IL-10 expression and suppressing proinflammatory cytokines. The detailed molecular mechanisms are described.

## Materials and Methods

### Clinical cohort

Patients with CRC enrolled in a clinical study investigating the CIPN (NCT02481336) were used for analysis. The details of this study are described elsewhere 19. Briefly, patients with pathologically confirmed stage III or high-risk stage II CRC have been prospectively recruited from National Cheng Kung University Hospital (NCKUH) from January 2015 until the present. All patients received curative surgery followed by 6 months of oxaliplatin and infusional 5-fluorouracil and leucovorin (modified FOLFOX6) as adjuvant chemotherapy. Patients with poor performance status (ECOG performance score > 1), inadequate organ functions, prior treatment with neurotoxic chemotherapy (such as oxaliplatin, cisplatin, carboplatin, taxanes, or vinca alkaloids), preexisting peripheral neuropathy of any grade, or a family history of genetic or familial neuropathy were excluded. A complete neurological examination, patients-reported peripheral neuropathy CIPN20 questionnaire (QLQ-CIPN20), and quantitative examination of the peripheral nerve system (including nerve conduction velocity [NCV], quantitative sensory test [QST], nerve excitability test [NET], and total neuropathy score [TNS]) were assessed at baseline, the end of cycles 1 (week 2), 6 (week 12), and 12 (week 24) of chemotherapy, and 9 (week 36) and 12 months (week 48) after the beginning of chemotherapy. The CIPN20 questionnaire contained 20 items evaluating the severity of neurologic symptoms, including nine items for sensory, eight items for motor, and three items for autonomic symptoms. Scores from 1 to 4 were applied for each item. The scores of the items were added together for all 19 questions, and a final CIPN score, ranging from 19 to 76, was generated. The score of item 20, which rates men's sexual function, was not adopted in this study because the information is lacking for women and is often not provided for male patients either. In addition to collecting blood from all participants at baseline to identify predictive biomarkers of CIPN, blood samples at week 48 were also collected in 23 patients to monitor the changes in biomarkers. This study was approved by the institutional review board of NCKUH (A-ER-103-395). All participants provided written informed consent before being enrolled.

### Reagents

Oxaliplatin was purchased from Sanofi Pharmaceutical Company (NY, USA). The procedure for synthesizing the CTSS inhibitor RJW-58 was described previously 14. The following primary antibodies were purchased: Cathepsin S (Thermo Fisher catalog: #PA5-81369), EBF (Santa Cruz, #sc-137065), ATF-3 (Biossua, #BS-0519R), α-tubulin (Sigma, MO, USA), IRF-1 (Bio-Rad, #VPA00801), CBP (GeneTex, #GTX101249), Iba1 (Abcam, #ab-5076), NeuN (Novus, #NBP2-10491), IL-10 (Abcam, #ab189392), STIM1 (Cell signaling (D88E10), #5668S), CD11b (Abcam, #ab52478), CD45 (BD Biosciences, #553079), STAT3 (BD, #50402), P-STAT3 (Cell signaling, #9145S), NFκB (Cell signaling, #4727S), OR5B3 (Abcam, #ab186624), OR5M3 (LSBio, #LS-C200406), and β-actin (GeneTex, #GTX109639). Horseradish-peroxidase-conjugated secondary antibodies were purchased from GeneTex (#GTX213110, #GTX213111, #GTX224125, and #GTX232040).

### Cell culture

DRG cell lines (ND 7/23) and mouse microglial cells (SIM-A9) were obtained from Sigma-Aldrich (#92090903, USA) and Kerafast (END001, USA), respectively. The ND 7/23 cells were grown in Dulbecco's Modified Eagle Medium (DMEM, #12100046; Gibco, USA) and the SIM-A9 cells were maintained in DMEM/F12 (#11320033; Gibco, USA) supplemented with 10% FBS, 100 U/ml of penicillin, and 100 μg/ml of streptomycin-glutamate (Thermo Scientific, Utah, USA). Both cell lines were maintained in a 5% CO_2_ incubator (humidified atmosphere) at 37 °C.

### Animals

The 6-week-old C57BL/6JNarl mice were maintained in the pathogen-free facility of the Animal Laboratory of National Cheng Kung University (Institutional Animal Care and Use Committee [IACUC] number: 107078). These animals were housed in a temperature-controlled (25 ± 2 °C); humidity of approximately (60%-80%) and light-controlled environment (12 / 12 h light-dark cycle, lights on at 6:00 AM) with free access to food and water, following the approved guidelines by the IACUC of National Cheng Kung University. Before the experiments were performed, all animals had adapted to the housing environments for at least 7 days. Our preliminary study revealed that no body weight loss was observed in animals treated with oxaliplatin 3 mg/kg or RJW-58 25 mg/kg intraperitoneally (*i.p.*) for 5 days a week over two alternative weeks.

### CTSS transgenic mice production

*Ctss*^-/-^ mice were generated using CRISPR/Cas9-induced knockout in embryos obtained from the National Laboratory Animal Center. Mouse fertilized eggs were microinjected with CRISPR/Cas9-related point mutation CTSS sequence, TGACTA, including a stop code and restriction enzyme SpeI recognition cutting site to verify the mutations at exons 2. The point mutation was designed right after the CTSS transcription start site to inhibit CTSS RNA and protein expression. Recombinant embryonic stem cell lines were injected into 161 C57BL/6JNarl blastocysts, 122 of which were transferred into pseudo‐pregnant females, and 28 live pups were born in 2018. Only one *Ctss*^+/-^ female strain was detected using polymerase chain reaction (PCR) genotyping. For recessive phenotypes, intercross between Ctss^+/-^ female mice with male Ctss^+/+^ mice in the related C57BL/6JNarl background generated Ctss^-/-^ mice. The litters were backcrossed three times to generate Ctss^-/-^ mice. Ctss^+/+^ C57BL/6JNarl mice were used as controls. The following primers were used for Ctss^-/-^ genotyping: forward 5′-GTCAGGCAGATTGCTACAAG-3′, reverse 5′-ACACTGCTCGGGTGGCAATC-3′, and digestion with restriction SpeI enzymes (Figure S1).

### Mouse behavioral models of oxaliplatin-induced peripheral neuropathy

Following housing adaptions, 7-week-old C57BL/6JNarl mice (weight range: 18-20 g) were used to investigate oxaliplatin-induced neuropathy. Oxaliplatin (3 mg/kg, Sanofi, USA), vehicle (saline), or RJW-58 (25 mg/kg) was administered *i.p.* for 5 consecutive days every other week for 2 weeks. A von Frey filament test (Part #2390, IITC Inc., CA) for mechanical hyperalgesia, tail immersion test (water temperature: 48-49 °C or ice) for thermal sensitivity, and grip test (BIO-EVF3, Bioseb Inc., USA) for grip strength were used, following a previously described method 11, 20. To quantify locomotor reactivity, we subjected each mouse to an open field chamber with photocell emitters and receptors equally spaced along the perimeter of the chamber. These photocell emitters and receptors created an x-y grid of invisible infrared beams and signals, recorded by computer software to calculate the quantity of mouse motor activity in 10 min. The baseline measurement of each behavior test was established before drug treatment, and five additional sessions were measured weekly.

### Primary DRG isolation

Primary DRG tissues were removed from adult C57BL/6JNarl mice after the behavior tests, and the tissues were digested with 0.1% collagenase for 1 h, followed by 0.25% trypsin for 25 min for cell mixture. The cell mixtures were stained with NeuN (Novus, #NBP2-10491) antibodies to isolate primary spinal dorsal horn neurons for flow cytometry analysis to detect ATF3 expression. The cell mixtures were further stained with both CD11b (Abcam, #ab52478) and CD45 (BD Biosciences, #553079) antibodies to isolate the microglia for flow cytometry analysis to detect IL-10 expression.

### Flow cytometry analysis

After treatment, the cells were fixed with 3.7% formaldehyde (dissolved in phosphate buffered saline [PBS]) for 15 min at room temperature and permeabilized with 0.5% Triton X-100 in 3.7% formaldehyde for another 15 min. The cells were washed three times with PBS and blocked with CAS-BlockTM solution (#008120; Life Technologies) for 1 h. The antibodies were prepared in the CAS-BlockTM solution (#008120; Life Technologies) and stained overnight. The cells were then washed three times with PBS and stained with secondary antibodies for 2 h. Finally, the cells were counterstained with DAPI for 2 min and then washed with 1-2 mL of BD FACSFlow buffer (LOT:1833202826) twice. After washing, the cells were resuspended with BD FACSFlow buffer. The data were acquired on a flow cytometer, following the manufacturer's recommendations.

### Cytokine array analysis

Ctss^+/+^ mouse serums were collected after RJW-58 injection (week 4). Cytokine array analysis was performed following the protocol of the MILLIPLEX_MAP_ mouse cytokine/chemokine magnetic bead panel (Millipore, MCYTOMAG-70K).

### Gene expression profiling and statistical analysis of microarray data

Total RNA samples were extracted using TRIzol (Invitrogen) and purified using a RNeasy mini-kit (QIAGEN). Gene expression analysis was performed on a GeneChip platform (Human Genome U133A 2.0 Plus; Affymetrix) following the National Health Research Institutes Microarray Core facility technique support. Gene expression changes were calculated as the ratio of 1.2-fold significant alterations of the experimental groups (CTSS overexpression or si-CTSS treated) compared with each control group. Compared with the gene expression variance within each experimental group, most of the olfactory receptor genes detected were differentially expressed.

### Ultrastructure of the sciatic nerve

After neurobehavioral tests at week 6 following oxaliplatin treatments, sciatic nerve samples were obtained from mice, fixed in 4% glutaraldehyde, and postfixed in 1% osmium tetroxide solution at 4 °C. These samples were then dehydrated in graded ethanol series and embedded in EMbed 812 (EMS-14120; Hatfield, PA, USA). The sciatic nerve sections (90 nm) were prepared by the Department of Pathology of NCKUH and observed and imaged using a transmission electron microscope (H7650, Hitachi, Japan).

### Quantification of intraepidermal nerve fiber density (IENF) density

Mouse hind paws were isolated after neurobehavioral tests. Dissected tissues were fixed with 10% paraformaldehyde overnight and cryoprotected for 72 h with 30% sucrose in PBS at 4 °C. The samples were embedded in OCT (#CM 3050S; Leica) and sliced into 30 μm thick sections. The slides were washed with 0.05% Triton X-100 in PBS for 4 h and incubated with CAS-Block^TM^ solution (#008120; Life Technologies) at room temperature for 1 h. The primary antibodies used were rabbit anti-PGP9.5 (1:100; #ab108986, Abcam) and goat anti-Collagens VI (1:100, #AB769; Chemicon). For double staining, two antibodies from different species were mixed and incubated overnight with the sample sections at 4 °C. After washing away the primary antibodies with PBS, the tissues were incubated with secondary antibodies (Alexa-488, 594, or 647, 1:200; Invitrogen) and Hoechst 33342 (10 mg/mL, #H1399; Invitrogen) for 2 h at room temperature. Immunofluorescence images were acquired using a multiphoton laser scanning microscope with a 40× objective (FV1000MPE, Olympus).

### Intracellular [Ca^2+^]_i_ measurement

ND7/23 or SIM-A9 cells (passage numbers: 10-15) were prepared and cultured for 3 days to measure intracellular Ca^2+^ concentration ([Ca^2+^]_i_). The cells were incubated with 2 μM Fluo-4/acetoxymethyl ester (Fluo-4/AM, F14201, Molecular Probes, USA) in DMEM medium at 37 °C for 30 min and then washed three times with Ca^2+^ free HEPES solution (145 mM NaCl, 5 mM KCl, 0 mM CaCl_2_, 10 mM glucose, 1 mM MgCl_2_, and 5 mM HEPES, with an osmolarity of 297-300, and pH 7.4). Fluo-4 was excited alternatively at 488 nm and the fluorescence intensity was analyzed at 520 nm. The Image XpressMicro automated wide-field fluorescent microscope (Molecular Devices, Sunnyvale, CA, USA) was then used to calculate [Ca^2+^]_i_. For [Ca^2+^]_i_ measurement, the cells were stimulated using 2 μM thapsigargin (TG, Cayman Chemical, USA), recorded for 7 min.

### Western blot

Cells were lysed with CelLytic MT Cell Lysis Reagent (Sigma) containing a protease inhibitor cocktail (Roche). The total cell lysates were separated with SDS-PAGE gel, transferred to polyvinylidene fluoride membranes (Amersham Life Science), blocked with 5% nonfat milk at room temperature for 1 h, washed three times with TBST (0.1% Tween-20), and then hybridized overnight with the primary antibody at 4 °C. The membranes were then washed three times with TBST (0.1% Tween-20) and hybridized with the appropriate peroxidase-conjugated secondary antibody (Santa Cruz) at room temperature for 1 h. Finally, the membranes were washed three times with TBST (0.1% Tween-20) and immunoreactivity was detected using Chemiluminescence (Millipore).

### Nuclear extraction assay

Samples were lysed using 500 μL of buffer A (10 mM HEPES, 1.5 mM MgCl_2_, 10 mM KCl, 0.5 mM DTT, and 0.05% NP-40; pH 7.9). After 10 min of centrifugation at 3,000 rpm at 4 °C, the supernatant was collected as a cytosolic fraction. The pellet was washed three times with a wash buffer (10 mM HEPES, 1.5 mM MgCl_2_, and 10 mM KCl; pH 7.9) and lysed using 450 μL of buffer B (5 mM HEPES, 1.5 mM MgCl_2_, 0.2 mM EDTA, 0.5 mM DTT, 26% glycerol, and 300 mM NaCl; pH 7.9). The supernatant was collected after 20 min of centrifugation at 13,200 rpm at 4 °C as a nuclear fraction. Finally, 100 μg of the cytosolic and nuclear fractions were subjected to gel electrophoresis and analyzed using immunoblotting.

### Coimmunoprecipitation and immunoblotting

Samples were lysed using an immunoprecipitation (IP) lysis buffer (0.5% NP-40 and 10% glycerol in PBS). A mixture of 2 mg of cell protein lysates, 5 μg of anti-STIM1 or anti-OLF1 antibodies, and 100 μL of protein-A/G sepharose beads were incubated overnight at 4 °C. The mixture was washed twice with wash buffer (0.5% NP-40, 0.1% Triton X-100 in PBS). The precipitated protein samples were processed for SDS-PAGE and detected using immunoblotting with the appropriate primary antibodies.

### Gene reporter assay

The reporter gene plasmids of CTSS-, IL10-pGL4.17 were purchased from GENEWIZ (product numbers: BB9283-1/N332028). The plasmids were introduced into cells using the oligofectamine transfection reagent for 24 h and then processed using the Dual-Luciferase 1000 Assay system (Promega, Madison, USA, E1910), following the manufacturer protocol.

### Chromatin immunoprecipitation PCR (ChIP)

The samples were crosslinked with 37% formaldehyde for 10 min at 37 °C. The cells were then harvested and processed for DNA fragmentation using sonication on ice. Following the manufacturer instructions, the resultant chromatin-DNA fractions were purified using a ChIP assay kit (Millipore, 2487694). The chromatin was immunoprecipitated at 4 °C using anti-IRF1, anti-OLF1, and normal IgG (negative control). The primer sequences used were as follows:

OLF1 on IL-10 promoter: forward 5′-CTGCCTTCATTATCAGTGTG-3′, reverse 5′-CAGCACAAAGCAAGATGTTG-3′; IRF1 on CTSS promoter: forward 5′-ATGCTCTATCTTTTCATCAGTCAG-3′, reverse 5′-GCCAACAAGAATCTCTCAAACAG-3′.

### Quantitative reverse transcription PCR

The total RNA was extracted from samples using TRIzol reagent (Thermo Fisher, #74108). First-strand cDNA was synthesized using the SuperScript III first-strand synthesis system following the manufacturer instructions. The transcription level of CTSS, IL-10, TNF-α, IL-1β, IL-6, and MCP-1 were quantified using the Applied Biosystems 7500 Real-Time PCR System, with RPL13A as an internal control. The primer sequences used were as follows: CTSS: forward 5′-CCATTGGGATCTCTGGAAGAAAA-3′, reverse 5′-TCATGCCCACTTGGTAGGTAT-3′; IL-10: forward 5′-GCTCTTACTGACTGGCATGAG-3′, reverse 5′-CGCAGCTCTAGGAGCATGTG-3′; MCP-1 Forward: 5′-AGGTCCCTGTCATGCTTCTG-3′, Reverse: 5′-GCTGCTGGTGATCCTCTTGT-3′; TNF-α Forward: 5'-AGTGACAAGCCCGTAGCCC-3', Reverse: 5'-AGCCTTGTCCCTTGAAGAG-3'; IL-1β Forward: 5'-GCTGAAAGCTCTCCACCTC-3', Reverse: 5'-GAGGTGCTGATGTACCAGTT-3'; IL-6 Forward: 5'-CAGGAAATTTGCCTATTGAAAAT-3', Reverse: 5'-TTGGATGGTCTTGGTCCTTAG-3'.

### Statistical analysis

At least seven mice were randomized to each group for all animal behavioral tests. The results are expressed as the mean ± SEM. Differences between groups were compared using a two-tailed Student's t-test or two-way analysis of variance, performed using Prism. Statistical significance was set as *P* < 0.05. Experimenters were blinded to all drug treatments.

## Results

### Evaluation of oxaliplatin-induced peripheral neuropathy in patients with colorectal cancer

**Figure 1:**
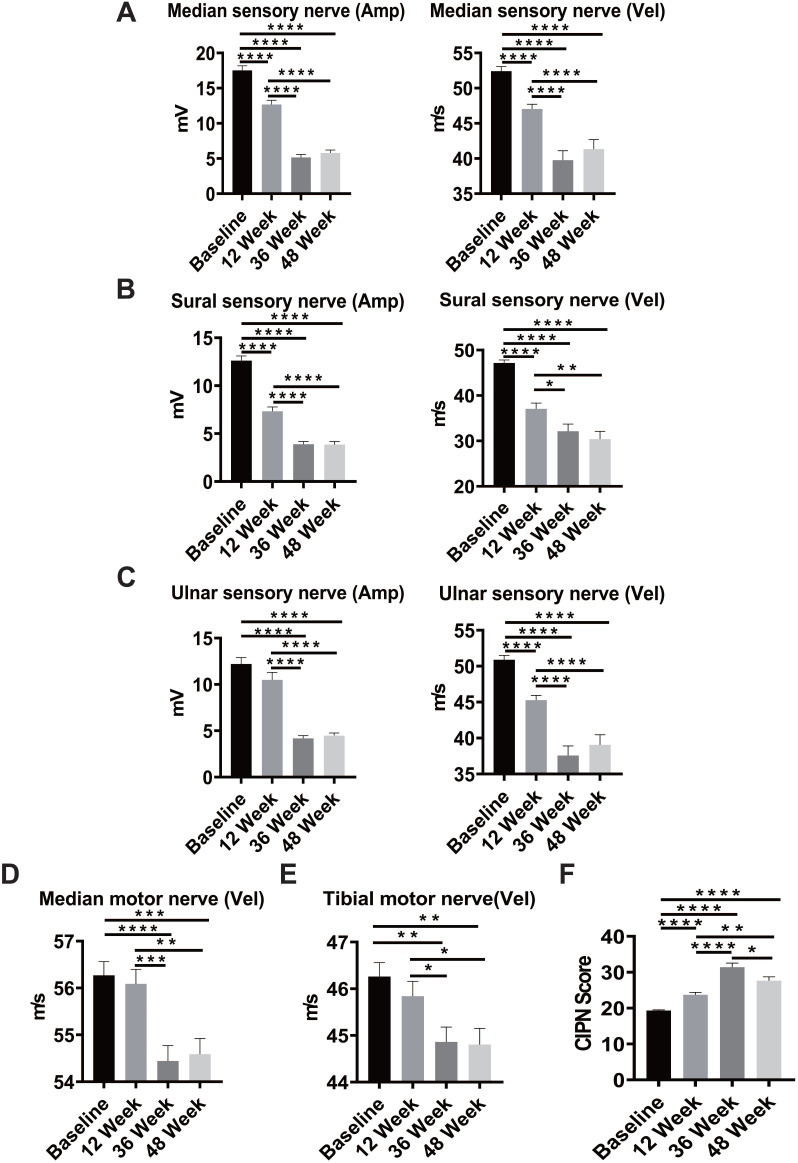
** Sensory and motor nerve deficit in patients with colorectal cancer (CRC) after oxaliplatin treatment.** A-C, Sequential measurement of the median, sural, and ulnar sensory nerve by NCV test before and after oxaliplatin treatment at baseline and weeks 12, 36, and 48 in CRC patients. D-E, Sequential measurement of median and tibial motor nerve before and after oxaliplatin treatment at weeks 12, 36, and 48 in CRC patients. F, The CIPN score at baseline and weeks 12, 36, and 48 in patients with colorectal cancer. ****P < 0.0001, ***P < 0.001, **P < 0.01, *P < 0.05 compared with baseline using a two-tailed Student's *t-test*. Each value represents the mean ± SEM from CRC patients.

From January 2015 to July 2019, 87 patients were recruited into a clinical study investigating chemotherapy-induced peripheral neuropathies: 45 (51.7%) men and 42 (48.3%) women. By July 2019, 76 of 87 patients had completed the 6-month adjuvant chemotherapy with modified FOLFOX6, and 75 patients (86.2%) had completed the neurologic study scheduled at week 12, 67 (77%) at week 36, and 63 (71.3%) at week 48. A significant reduction in both amplitude and conduction velocity was observed in sensory nerves, including the median sensory nerve (Figure 1A), sural sensory nerve (Figure 1B), and ulnar sensory nerve (Figure 1C), after 6 cycles of chemotherapy (week 12) compared with the baseline results of nerve conduction (*P* < 0.0001). The slowing in conduction velocities and the reduced amplitude deteriorated further at week 36 and reached a plateau at week 48, which was 6 months after completion of the oxaliplatin-containing chemotherapy. Peripheral sensory neuropathy is a well-known toxicity of oxaliplatin. However, motor impairments have been less well characterized. Notably, we observed a similar pattern of reduction in conduction velocity in median motor nerves (*P* < 0.0001) and tibia motor nerves (*P* = 0.0015, *P* = 0.0018; Figure 1D-E). The CIPN20 questionnaire was used to assess patient-reported severity of peripheral neuropathy, and the CIPN scores increased significantly from baseline to week 12, suggesting an increase in neurologic symptoms after six cycles of chemotherapy (Figure 1F). The CIPN20 scores reached a peak at week 36 and declined significantly at week 48 (*P* < 0.0001). These results indicate that oxaliplatin-containing chemotherapy-induced subjective and objective sensory and motor neuropathies in CRC patients.

### Targeting cathepsin S alleviates oxaliplatin-induced peripheral neuropathy *in vitro* and *in vivo*

**Figure 2:**
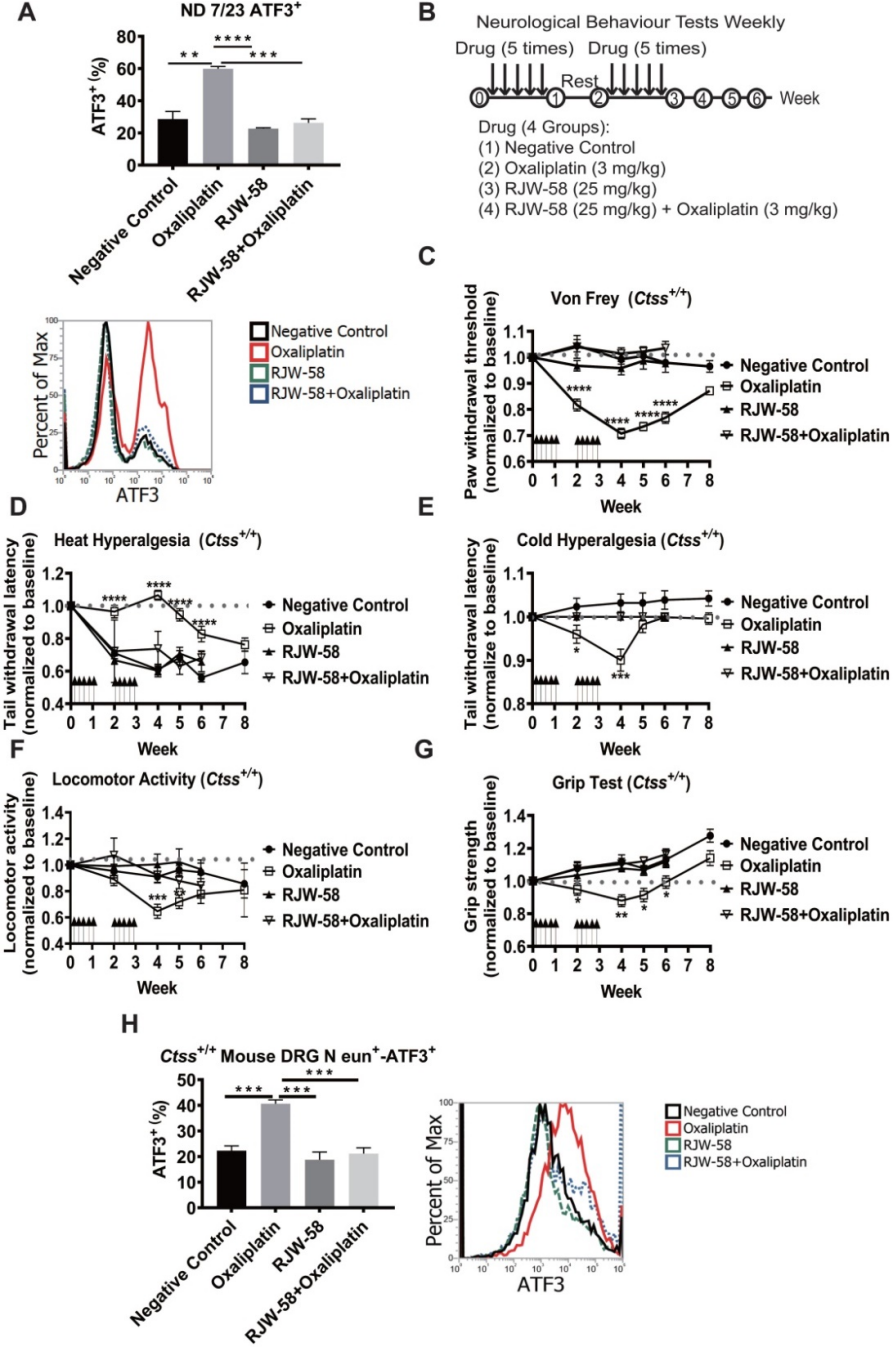
** The neuropathic effects of oxaliplatin in the* Ctss*^+/+^ mouse model.** A, Flow analysis of ATF3 expression in ND 7/23 cell treated with control, oxaliplatin (1 µM), RJW-58 (5 µM), or a combination of oxaliplatin (1 µM) and RJW-58 (5 µM), respectively. B, The protocol of drug administration and behavioral tests in the mouse model. The basal levels of each behavioral assay were obtained before treatment. In the first week, PBS control (100 µL), oxaliplatin (3 mg/kg, 100 µL), and RJW-58 (25 mg/kg, 100 µL) were injected intraperitoneally five times per week for 2 weeks. Behavioral tests were performed weekly. C, Von Frey filament test to detect the paw withdrawal threshold to mechanical stimulus. The Y-axis is the normalized pressure from touch to paw withdrawal. D-E, Tail immersion test to assess thermal sensation. The Y-axis is the normalized latency from tail immersion to tail withdrawal. F, Locomotor activity test to detect allodynia. The Y-axis is the normalized moved distance baseline. G, Grip test to detect neuromuscular function. The Y-axis is the normalized moved distance baseline. The black arrow indicates drug infusion. H, Flow analysis of ATF3 expression of primary DRG cells at week 4 for NeuN^+^-ATF3^+^ double staining. ****P < 0.0001, ***P < 0.001, **P < 0.01, *P < 0.05 compared with baseline using a two-tailed Student's t-test. Each value is represented as mean ± SEM from at least seven mice in each group.

We had previously successfully established an integrated image-based high-content screening platform to identify neuroprotective compounds 11, 20. By applied the high-content drug screening platform, several compounds were tested for protective effects against chemotherapy-induced neurotoxicity, including cathepsin S inhibitors. Furthermore, our studies revealed that inhibition of cathepsin S by RJW-58 could retard tumor progression and metastasis 14. Importantly, similarly with previous findings, elevated levels of CTSS are associated with increased risk in the development of neuropathic pain 21, 22. And, blocking CTSS activity could relieve inflammatory hyperalgesia 23, 24. Above evidence suggested that cathepsin S inhibitors, as potential compounds, prevent chemotherapy-induced neurotoxicity. Therefore, we investigated whether cathepsin S inhibitor (RJW-58) could prevent oxaliplatin-induced neurotoxicity *in vitro*. Our results indicated that RJW-58 suppressed oxaliplatin-induced ATF3 expression in ND 7/23 cells (Figure 2A). ATF3 is a marker of damaged neurons 25.

To validate the neuroprotective effects of RJW-58 *in vivo*, C57BL/6JNarl-*Ctss*^+/+^ mice were treated with either oxaliplatin, RJW-58, or both (Figure 2B). Mouse behavioral tests, including tail immersion, von Frey filament, locomotor activity, and grip tests, were then performed weekly to assess the thermal threshold, mechanical allodynia, motor function, and fine motor skills, respectively. The oxaliplatin-treated mice exhibited a dramatic reduction in mechanical thresholds (Figure 2C) and an increase in thermal thresholds (Figure 2D), which was consistent with the clinical neurological findings for CRC patients treated with oxaliplatin (Figure 1). Notably, cold hyperalgesia recovered gradually, 2 weeks after the cession of oxaliplatin treatment, and returned to baseline at week 6 (Figure 2E). Furthermore, the locomotor activity and grip strength were also dramatically reduced by oxaliplatin treatment (Figure 2F-G), but recovered gradually to baseline at week 6. The *post-hoc* analysis data obtained at post-treatment week 6 revealed a significant protective effect of RJW-58 on oxaliplatin-induced sensory and motor deficits. Moreover, we isolated the primary DRG cells from *Ctss*^+/+^ mouse spinal cord before and after drug treatment. Consistent with the results from ND 7/23 cell lines, oxaliplatin treatment enhanced ATF3 expression, and RJW-58 reversed the effect of oxaliplatin on mouse DRG cells (Figure 2H).

*Ctss*^-/-^ mice were generated to confirm that CTSS plays a critical role in protecting neurons from oxaliplatin damage (Figure S1A). Western blot analysis confirmed no expression of CTSS in *Ctss*^-/-^ mouse (Figure S1B). Mice were then treated with the same dose and schedule of oxaliplatin, as illustrated in Figure 2B, and neurobehavioral tests were then performed. No differences were observed in sensory and motor function test results between *Ctss*^-/-^ mice treated with or without oxaliplatin (Figure S2A-E). Together, these data indicate that CTSS plays a pivotal role in the development of OIPN.

### Histology of sciatic nerve and quantification of intraepidermal nerve fiber density

**Figure 3:**
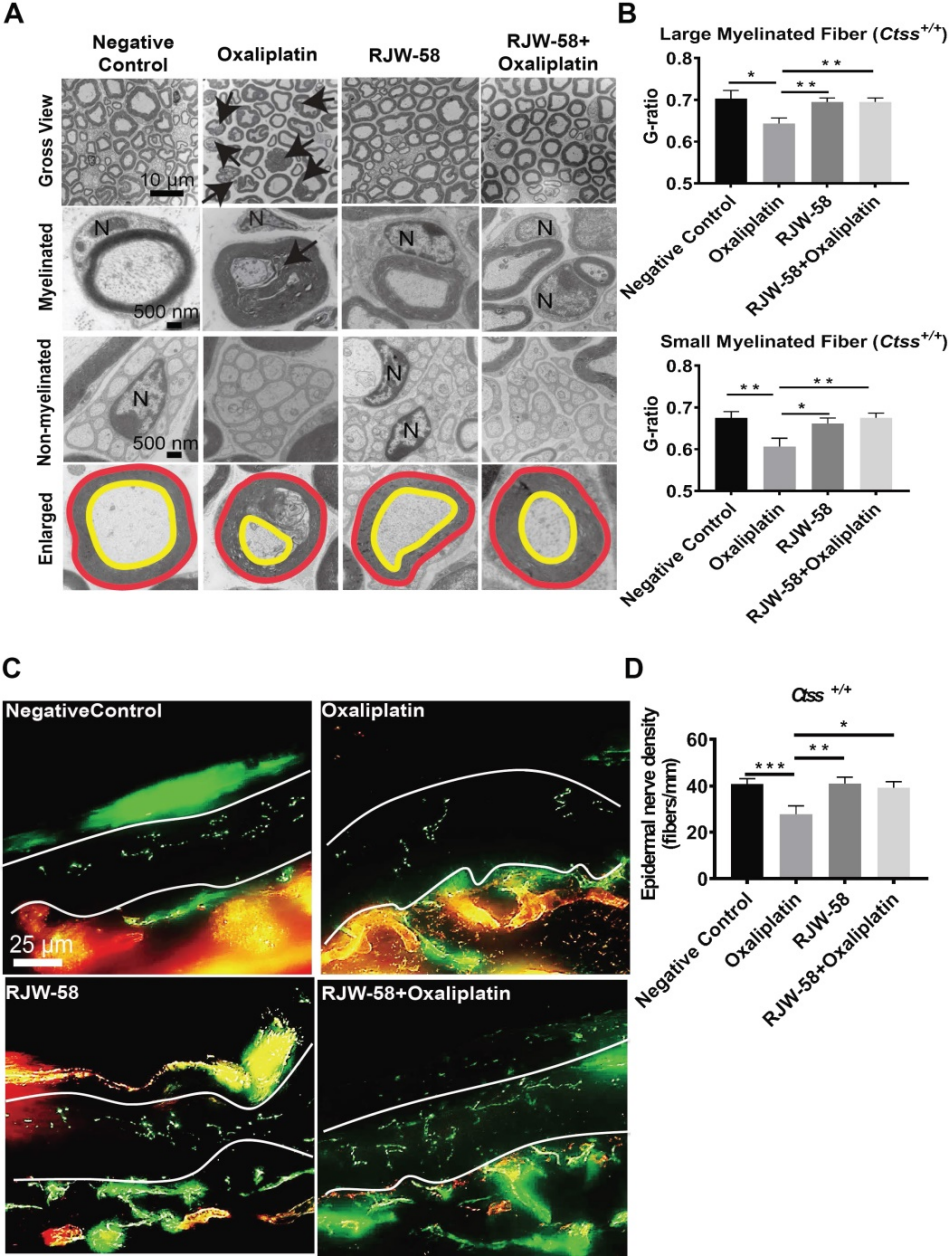
** RJW-58 protects peripheral nerves from oxaliplatin-induced damage.** A, Transmission electron micrographs of the sciatic nerve from *Ctss^+/+^* mice treated with control, oxaliplatin, RJW-58, or the combination of oxaliplatin and RJW-58. Bottom: G-ratio's measurement represents the ratio of axon diameter (yellow line) to myelin (red line). N: nucleus of Schwann cell. Scale bar: 10 µm (top), 500 nm (middle and bottom). B, The myelinated fiber density of sciatic nerves from mice in different treatment groups was measured at week 6 (n = 5 mice/group). The G-ratio was calculated and compared between different treatment groups. C-D, Representative immunofluorescence images and quantitative analysis of intraepidermal nerve fibers of mice treated with control, oxaliplatin, RJW-58, and a combination of oxaliplatin and RJW-58. Scale bar: 25 µm. ****P < 0.0001, ***P < 0.001, **P < 0.01, *P < 0.05 compared with baseline using a two-tailed Student's t-test. Each value represents the mean ± SEM from at least 7 mice in each group.

Two methods were used to examine histopathological differences before and after oxaliplatin treatment. First, we inspected the ultrastructure of sciatic nerves using a transmission electron microscope at week 6 after drug treatment. Our data demonstrated that oxaliplatin damaged both myelinated and nonmyelinated nerve fibers. These fibers exhibited varying degrees of axon death (black arrow), axons with detached compact myelin, and accumulated myelin debris. In contrast, RJW-58 cotreatment reduced oxaliplatin-induced nerve damage (Figure 3A). Furthermore, we assessed the integrity of the myelinated nerve by calculating the ratio of inner axon circumference to outer myelin circumference (G-ratio). As depicted in Figure 3B, oxaliplatin treatment significantly reduced the G-ratio of both large myelinated (axon diameter > 5 μm; *P* = 0.01) and small myelinated fibers (< 5 μm; *P* = 0.008) compared with the control, whereas RJW-58 significantly reduced the axonal damage (*P* = 0.003).

Second, morphologic changes in skin innervations are associated with clinical measures of small-fiber neuropathy and have proposed that oxaliplatin could induce intraepidermal nerve fiber (IENF) loss 26. Therefore, IENF density in the mouse hind-paw interplanar skin was calculated before and after oxaliplatin treatment with or without RJW-58. As illustrated in Figure 3C, oxaliplatin treatment caused 68% IENF loss in the interplanar epidermis (Figure 3D; *P* = 0.003). However, no differences in IENF density were observed between oxaliplatin-treated mice co-treated with RJW-58 and control mice.

Furthermore, *Ctss*^-/-^ mice were also used to examine the neuroprotective role of CTSS in oxaliplatin-induced nerve damage. Our results indicated that no differences in ultrastructure changes in the sciatic nerve or the number of IENF between oxaliplatin-treated and control mice (Figure S2F-I). Furthermore, no differences were revealed in the expression level of ATF3 in microglial cells isolated from DRG in mice treated with or without oxaliplatin (Figure S2J). These findings further confirm the neuroprotective effect of CTSS inhibition in OIPN.

### Oxaliplatin treatment increases CTSS expression and binding to STIM1

**Figure 4:**
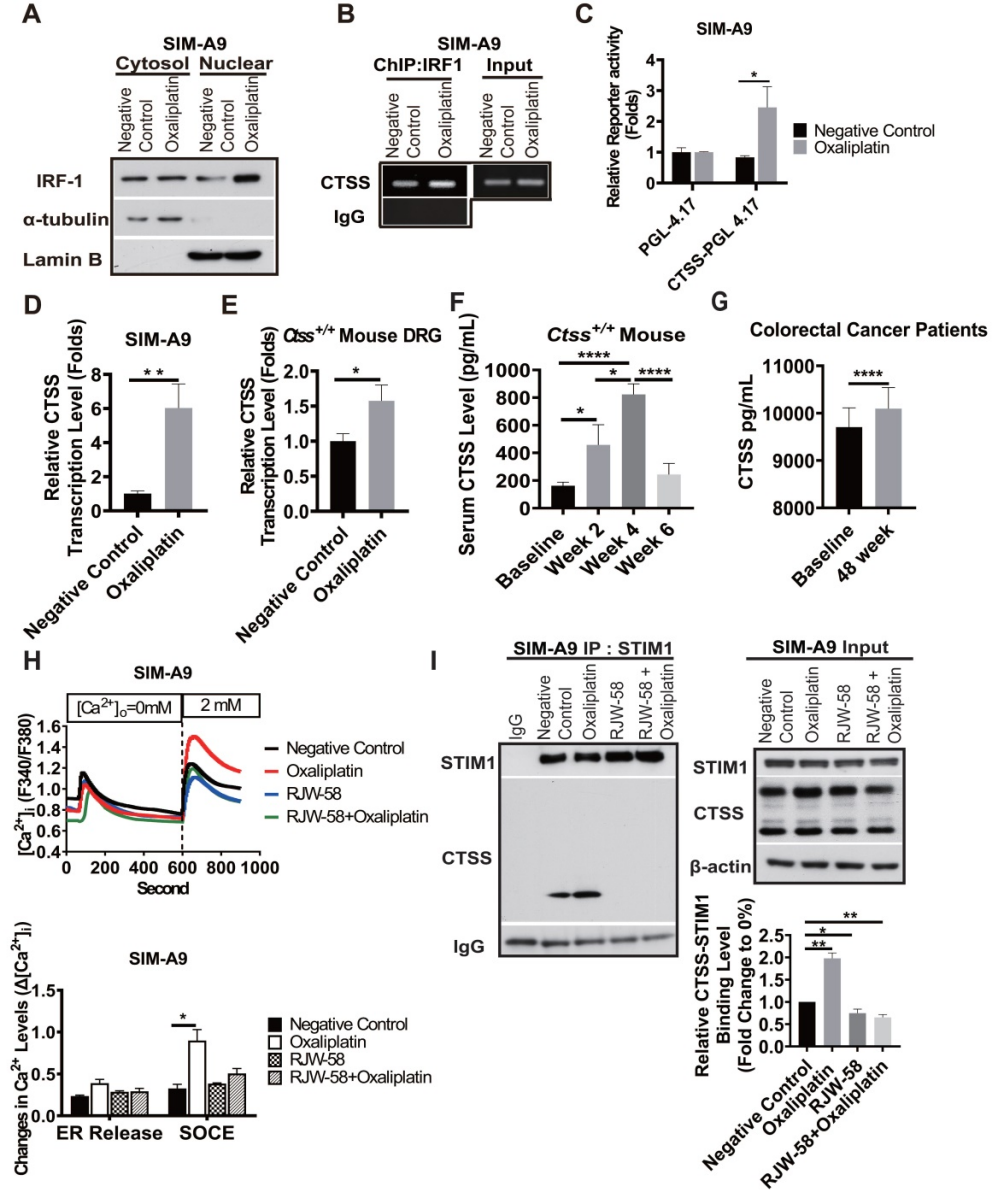
** Effects of oxaliplatin-induced remodeling of calcium homeostasis on CTSS expression via IRF1.** A, The effect of oxaliplatin on the nuclear localization of IRF1. Cells were treated with or without oxaliplatin (1 µM) for 24 h, and the cytosolic and nuclear fractions were then separated, as described in the Materials and Methods section. B, The effect of oxaliplatin treatment for 24 h on IRF1 binding to the CTSS promoter regions in SIM-A9 cells was analyzed using ChIP. C, The effect of oxaliplatin treatment for 24 h on the transcriptional activity of CTSS promoters was determined using the promoter assay. D, The effect of oxaliplatin treatment for 24 h on the transcription levels of CTSS genes in SIM-A9 was determined using RT-qPCR. E, The effect of oxaliplatin on the transcription levels analysis of CTSS genes in mouse primary DRG at week 4 was determined using RT-qPCR. F, The time-dependent mouse serum CTSS level (n = 10). G, Patients with colorectal cancer' serum CTSS level at week 48 compared to baseline (n = 23). ****P < 0.0001, Two-way ANOVA analysis. H, For the drug treatment, the cells were pretreated with DMSO or RJW-58 (5 µM). The treatment time for ND 7/23 and SIM-A9 was 24 h. The calcium influx from the ER and SOCE in CTSS overexpression (n = 50 for each condition) cell was assessed using single-cell intracellular Ca^2+^ measurement. I, CTSS was disassociated from STIM1 following RJW-58 treatment. ****P < 0.0001, ***P < 0.001, **P < 0.01, *P < 0.05. The statistical significance was determined using a two-tailed Student's t-test.

DNA damage caused by drugs, such as cisplatin and doxorubicin, results in the upregulation of interferon regulatory factor 1 (IRF1) 27. A study revealed that CTSS expression is regulated by transcriptional factor IRF1 28. Therefore, we determined whether oxaliplatin could enhance CTSS expression by regulating IRF1 in SIM-A9 cells. Our data demonstrated that oxaliplatin treatment increased cytosol translocation of IRF1 to the nucleus (Figure 4A) and enhanced IRF1 binding to the CTSS promoter region, which augmented the transcriptional level of CTSS *in vitro* and *in vivo* (Figure 4B-E).

We further investigated whether serum CTSS level could serve as a predictive biomarker of oxaliplatin-induced peripheral neuropathy. Our data revealed that the serum level of CTSS in mice increased at week 2 (*P* = 0.049), peaked at week 4 (*P* < 0.0001), and returned to baseline level at week 6 after oxaliplatin treatment (Figure 4F). These changes were consistent with findings that sensory and motor function started to decline at week 2, reached their nadir at week 4, and recovered at week 6 after oxaliplatin treatment, as illustrated in Figure 2. Furthermore, we retrospectively verified serum CTSS level in 23 patients at week 48 (Figure 4G) compared for the nerve conduction velocity test. Consistent with animal models' findings, motor function recovers more rapidly than sensory function (Figure 2).

Disruption of store-operated calcium entry (SOCE) through STIM1 has been reported that also underlies chemotherapy-induced neuropathy 11. Our previous study demonstrated that CTSS plays a crucial role in mediating Ca^2+^ homeostasis by binding to STIM1, thus interfering with Orail1 14. Therefore, we investigated whether oxaliplatin augments CTSS expression results in the dysregulation of Ca^2+^ homeostasis and also examined whether inhibiting CTSS enzyme activity may restore the SOCE disruption. Compared to control group, oxaliplatin significantly increased SOCE Ca^2+^ level after thapsigargin-induced endoplasmic reticulum (ER) store depletion (Figure 4H, *P* = 0.04). RJW-58 treatment alone did not affect the SOCE activation. However, co-treated with oxaliplatin, blocking CTSS by RJW-58 prevented oxaliplatin-induced SOCE dysregulation. Our previous study had reported that CTSS inhibition reduced Ca^2+^ influx through SOCE, but did not affect STIM1 and Orai 1 expression 14. Hence, immunoprecipitation assays were performed to confirm whether oxaliplatin evoked SOCE disruption via the augmentation of CTSS-STIM1 binding affinity. Our results demonstrated that CTSS bound to STIM1, which was enhanced by oxaliplatin treatment in SIM-A9 cells and reduced by cotreated with RJW-58 (Figure 4I).

### RJW-58 treatment induces anti-inflammatory cytokine production and suppresses proinflammatory cytokine production

**Figure 5:**
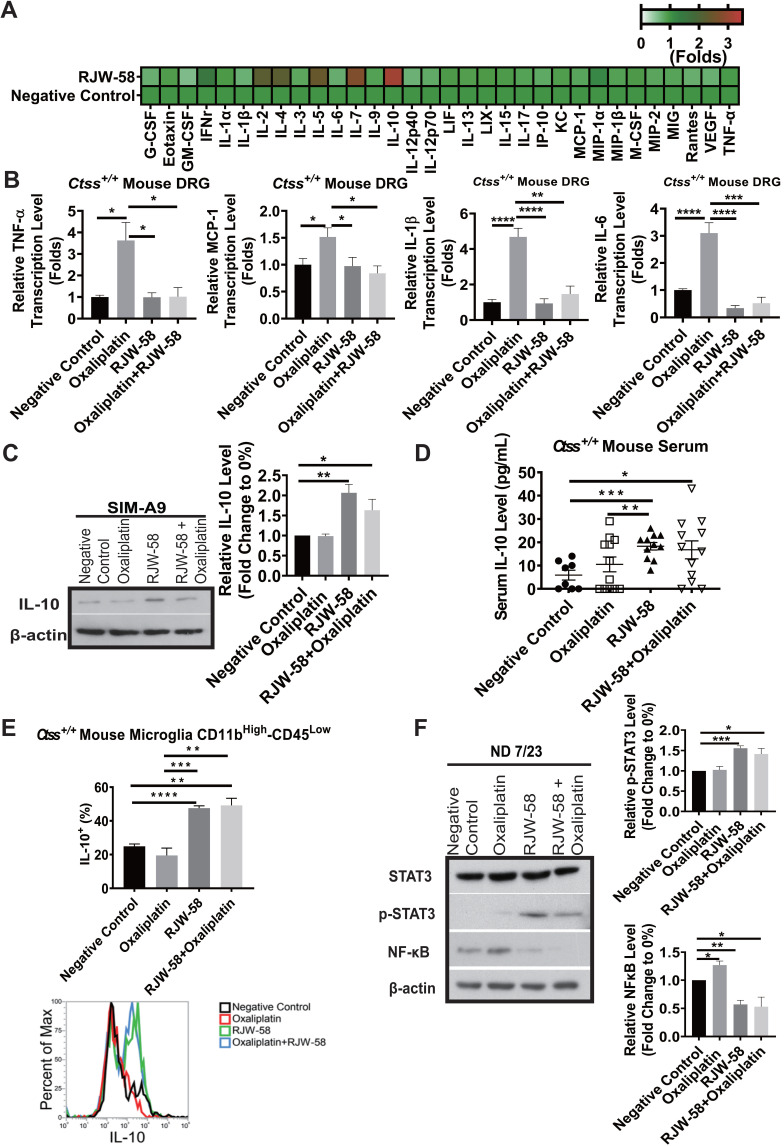
** Effects of RJW-58 in IL-10 expression *in vitro* and *in vivo*.** A, Heat map of cytokine array clustering analysis. RJW-58 increased IL-10 secretion in B6 mouse serum at week 4. B, The effect of RJW-58 (5 µM) in primary DRG cells on the transcription levels of TNF-α, IL-6, IL-1β, and MCP-1 genes at week 4 was determined using RT-qPCR. C, Western blotting of IL-10 expression level in SIM-A9 after RJW-58 for 24 h treatment. D, The mouse serum IL-10 level after RJW-58 treatment at week 4. E, Flow analysis of CD11b**^+^**-CD45**^-^** B6 primary microglial cells at week 4 for IL-10 expression. F, Western blotting of the NF-κB/STAT3 signaling pathway under treatment with RJW-58 for 24 h. ****P < 0.0001, ***P < 0.001, **P < 0.01, *P < 0.05. The statistical significance was determined using a two-tailed Student's *t-test.* Data are presented as the mean ± SEM.

Neuroinflammation is a pathological feature of CIPN, and consequently, therapeutics targeting the suppression of immune response has become an amenable strategy for CIPN. Furthermore, our data indicated that inhibition of CTSS reduced intracellular Ca^2+^ influx, which was reported to initiate macrophage activation 15, 29. Therefore, investigating cytokine profiles before and after CTSS inhibition is reasonable. We then collected mouse serums at week 4 after RJW-58 *i.p*. injection and analyzed the cytokine profiles using a cytokine array. Our findings indicated that RJW-58 dramatically increased anti-inflammatory cytokine expression, with a predominant IL-10, IL-5, and IL-4 expression and a concomitant reduction in proinflammatory cytokine expression, including TNF-α, IL-6, IL-1β, and MCP-1 (Figure 5A). The transcriptional levels of TNF-α, MCP-1, IL-1β, and IL-6 in mouse DRG were increased significantly after oxaliplatin treatment, which was reversed by RJW-58 (Figure 5B). We focused on CTSS and IL-10 because of the pleiotropic immunosuppressive function of IL-10. We determined that translational levels of IL-10 increased significantly in SIM-A9 cells treated with either RJW-58 alone or cotreated with oxaliplatin (Figure 5C). Furthermore, the serum level of IL-10 in RJW-58-treated* Ctss^+/+^* mice was significantly higher than the animals with vehicle or oxaliplatin alone (Figure 5D; *P* = 0.0002 and *P* = 0.0042, respectively). Moreover, the serum level of IL-10 in* Ctss^-/-^* mice was significantly higher than in *Ctss^+/+^* mice (Figure S2K, *P* < 0.0001). Furthermore, a similar result was obtained in microglial (CD11b^+^-CD45^-^) cells isolated from mice (Figure 5E). IL-10 was reported to exert its function by activating the IL-10/STAT3 anti-inflammatory pathway cascade. In contrast to increasing NF-κB levels after treatment with oxaliplatin in ND 7/23 cells, RJW-58 treatment alone or cotreatment with oxaliplatin increased p-STAT3 and reduced NF-κB expression levels (Figure 5F). Our finding supported that CTSS inhibition results in an anti-inflammatory response in oxaliplatin-induced neuropathy, through the upregulation of IL-10.

### CTSS is involved in the regulations of the olfactory transcription factor, Olf-1

**Figure 6:**
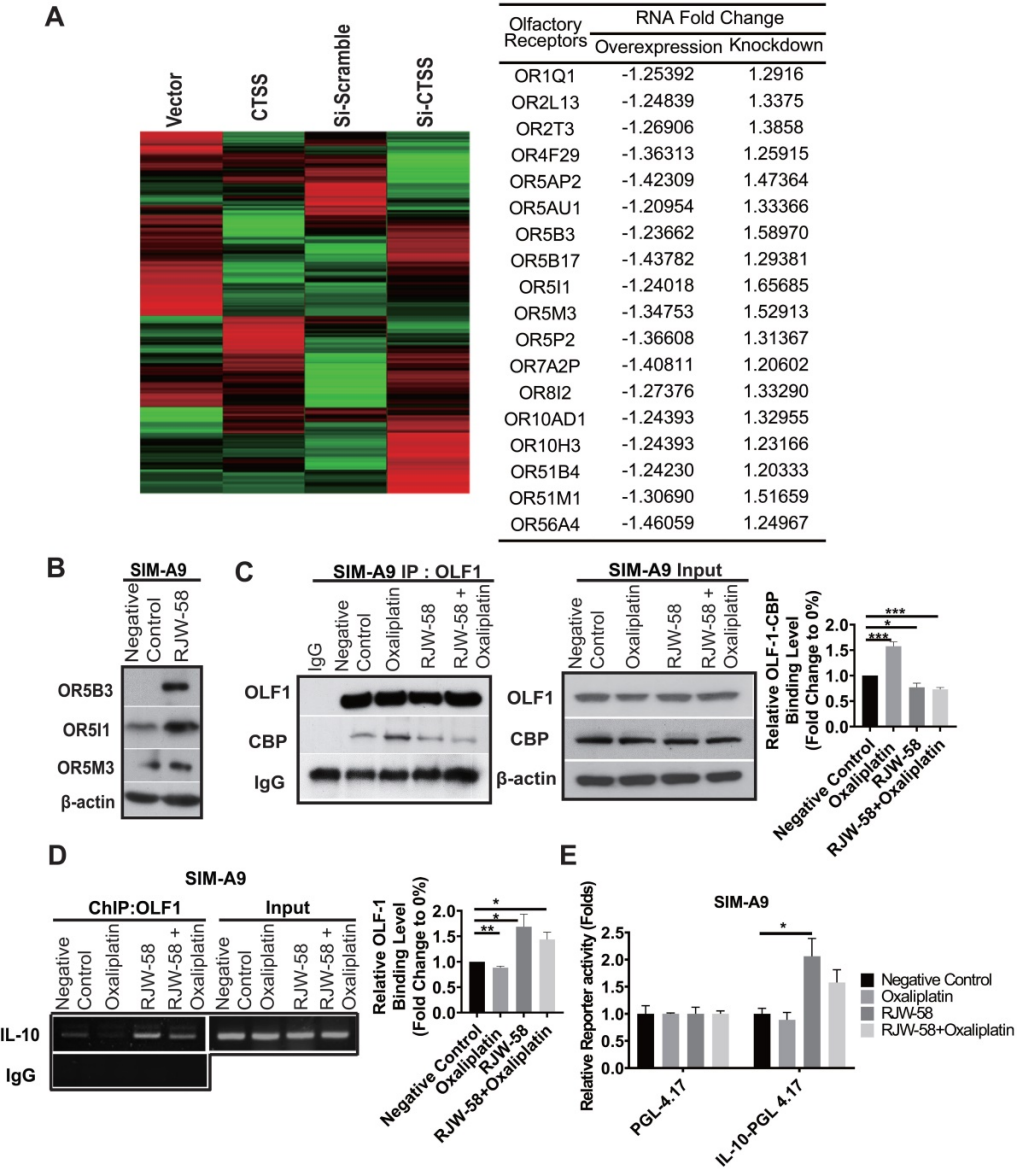
** Effects of RJW-58 in IL-10 expression via OLF-1.** A, The cluster heat map reveals the results of microarray analysis. CTSS knockdown increases the mRNA levels of the olfactory receptor family. B, Western blot analysis of OR5B3, OR5I1, and OR5M3 proteins when SIM-A9 cells were treated RJW-58 (5 µM) for 24 h. C, OLF-1 disassociated with CBP by RJW-58 for 24 h treatment in SIM-A9 cells. D, The effect of RJW-58 for 24 h on OLF-1 binding to the IL-10 promoter region in SIM-A9 cells was analyzed using ChIP. E, RJW-58 treatment facilitated IL-10 promoter activity in SIM-A9. IL-10 promoters were identified using the promoter assay. ****P < 0.0001, ***P < 0.001, **P < 0.01, *P < 0.05. The statistical significance was determined using a two-tailed Student's t-test. Data are presented as the mean ± SEM.

Previously, we investigated genes that may be regulated by the expression of CTSS. The siRNA knockdown and overexpression by transient transfection with CTSS in cancer cell lines was performed and applied to gene expression profiling. Notably, microarray results revealed that CTSS knockdown upregulated almost all olfactory receptor family genes, and* vice versa*, in cell overexpressing CTSS (Figure 6A). Western blot analysis confirmed the increase of the expression level of olfactory receptor 5B3, 5I1, and 5M3 proteins when SIM-A9 cells were treated with RJW-58 (Figure 6B). Evidence has indicated that olfactory receptors are regulated by an olfactory receptor transcription factor, Olf-1, which was reported that CBP competes with Olf-1 to bind DNA and activate target genes 30. Above finding suggested that inhibition of CTSS might affect the interaction between CBP and Olf-1, which results in the release of Olf-1 and binding to the target genes. Oxaliplatin treatment increased the recruitment of p300/CBP and Olf-1, which was reduced when cells were treated with RJW-58 alone or combined with oxaliplatin (Figure 6C). Next, the ChIP experiment demonstrated that incubation with oxaliplatin decreased Olf-1 transcription factor binding to IL-10 promoter. RJW-58 alone or co-treatment with oxaliplatin promoted released Olf-1 from p300/CBP for binding to the Olf-1-binding sites on the IL-10 promoter regions (Figure 6D). We further performed a luciferase functional quantitative assay to assess the effects of RJW-58 on IL-10 promoter activity. Comparison of the PGL-4.17 vector, RJW-58 promotes a two-fold increase in IL-10 transcriptional activity (Figure 6E, P = 0.04). These findings indicate that oxaliplatin treatment facilitated the assembling of p300/CBP and Olf-1. By contrast, inhibiting CTSS by RJW-58 decreased the recruitment of CBP and Olf-1. These released Olf-1 transcription factors further bind to Olf-1-binding sites on the IL-10 promoter regions (Figure 6D), which results in an increased IL-10 transcriptional activity (Figure 6E).

## Discussion

Oxaliplatin, a platinum derivative, is widely used as first-line adjuvant therapy in advanced CRC 31. Oxaliplatin can cause acute pain syndromes, which usually subside within days after cessation of treatment, and chronic distal neuropathy, which occurs after several cycles of treatment 32. However, less is known regarding oxaliplatin-induced motor impairments from clinical studies or animal models. Our clinical study demonstrated that oxaliplatin could cause both sensory and motor impairments in patients receiving a standard dose and course of adjuvant chemotherapy with an oxaliplatin-containing regimen after the definitive operation (Figure 1). Furthermore, despite numerous animal models having been established to understand the mechanisms and explore the therapeutic strategies to treat or prevent oxaliplatin-induced peripheral neuropathy, most of these models were not fully relevant to clinical findings. Therefore, establishing an appropriate animal model is warranted. By administering oxaliplatin according to the treatment protocol illustrated in Figure 2B, we demonstrated that oxaliplatin treatment caused both sensory and motor neuropathies without reducing body weight in the mice model. Furthermore, oxaliplatin treatment significantly reduced the G-ratio of both large myelinated and small myelinated fibers, compared with the control, assessed using electron microscopy (Figure 3). These animal data further support that oxaliplatin can cause both sensory and motor impairments, which is consistent with clinical findings (Figures 1 and 2). Discrepancies between the findings in this study and other studies are mainly caused by different dosing schedules and cumulative doses of oxaliplatin 33, 34. We demonstrated, using this animal model, that CTSS inhibition could protect animals from oxaliplatin-induced neuropathy, which was supported by the results from mouse behavioral tests, EM studies of sciatic nerves, and quantification of IENF density (Figures 2 and 3). The pivotal role of CTSS in oxaliplatin-induced neuropathy was further validated in the *Ctss^-/-^* knockout mouse model (Figure S1).

Lines of evidence have demonstrated that the ubiquitous second messenger Ca^2+^ plays a crucial role in fundamental physiological functions 35, including cell motility, apoptosis, cell cycle control, and gene expression. Under inflammations, dysregulation of intracellular Ca^2+^ homeostasis is a vital mechanism for regulating neurite outgrowth and subsequent neurite damage 36. Our previous studies have revealed that SOCE components participate in modulating calcium homeostasis in response to inflammation and partly contribute to paclitaxel-induced neuropathies, which were also determined in the present study 11, 20. A study demonstrated that oxaliplatin was degraded to oxalate and platinum inside the cell. Oxalate by chelating intracellular Ca^2+^ disrupts neuronal membrane potentials and further dysregulates the activity of voltage-gated ion channels. However, these disruptions only caused cold allodynia in mice 37, 38. Furthermore, oxaliplatin treatment was reported to induce intracellular Ca^2+^ influx, but the mechanism remains unclear 39. Our previous study has shown CTSS inhibition reduced Ca^2+^ influx through affecting SOCE activation and interfering STIM1 trafficking, which leads to the suppression of tumor cell progression. Furthermore, immunofluorescence data further demonstrated that CTSS could bind to STIM1, which was reversed by CTSS inhibitor. This effect was caused by accumulating STIM1 puncta in the ER, thereby reducing the interaction between active STIM1 and EB1 14. In the present study, we demonstrated that oxaliplatin treatment significantly increased the SOCE Ca^2+^ level after thapsigargin-induced ER store depletion, which was reversed by CTSS inhibition with RJW-58 (Figure 4H). Notably, we determined that oxaliplatin treatment increased CTSS expression by promoting IRF1 translocation from the cytosol to the nucleus, thereby enhancing IRF1 binding to the CTSS promoter region, which amplified the transcriptional level of CTSS (Figure 4A-E). Furthermore, chemotherapeutics stimuli may cause alterations of lysosomal membrane permeability and leakage of cathepsins into the cytoplasm 28. These data explain why oxaliplatin could increase CTSS expression, causing an increase in binding to STIM1 (Figure 4I), indicating that the regulation of CTSS expression contributes to the effect of oxaliplatin on intracellular Ca^2+^ homeostasis.

Neuroinflammation is a hallmark of the pathogenesis of CIPN. Increasing evidence demonstrates that excessive inflammation in the damaged nervous tissue after chemotherapy contributes to the initiation and maintenance of peripheral neuropathies 36. Oxaliplatin treatment increased the production and release of TNF and IL-1β through the upregulation of P2X purinoreceptor 7 in human neuroblastoma cell lines 40. Furthermore, *in vivo* study revealed that oxaliplatin treatment causes an increased number of activated satellite glial cells in the spinal cord 41. CTSS is reportedly an autocrine factor that stimulates microglial cell activation and subsequently regulates the release of proinflammatory cytokines and chemokines 13. In the present study, we demonstrated that CTSS inhibition with RJW-58 increases the serum levels of anti-inflammatory cytokines (predominantly IL-10, IL-4, and IL-7) and suppresses proinflammatory cytokines (Figure 5A). This effect was further validated in an animal model, which demonstrates that oxaliplatin treatment results in the upregulation of TNF-α, IL-6, IL-1β, and MCP-1 in mice DRG cells, which is suppressed by cotreated with RJW-58 (Figure 5B). Studies have reported that exogenous administration of the anti-inflammatory cytokine IL-10 suppresses allodynia in surgery 9, 10 and chemotherapy-induced neuropathy 42. Furthermore, increasing evidence suggests that STIM-dependent Ca^2+^ influx is required for IL-10 production because dysregulation of Ca^2+^ homeostasis impairs IL-10 secretion 43.

In this study, we determined that RJW-58 reversed oxaliplatin-induced dysregulation of Ca^2+^ homeostasis and increased endogenous IL-10 by activating the transcription factor Olf-1. We discovered that Olf-1 and transcriptional coactivators CBP coregulated the induction of IL-10 genes. Studies have indicated that the HAT activity of p300/CBP is involved in regulating transcription by remodeling chromatin, which is inhibited by binding with Olf-1 30, 44. In the ChIP experiment, oxaliplatin treatment facilitated the assembling of p300/CBP and Olf-1, whereas RJW-58 reversed this assembling and further drove IL-10 gene transcription by activating Olf-1. Furthermore, the elevation of endogenous IL-10 after RJW-58 treatment sequentially triggers the IL-10/STAT3 cascade. IL-10 is also involved in raising feedback inhibition, which limits the inflammation cascade. Therefore, IL-10 decreases NF-κB expression, which causes a reduction in the induction of inflammation in mouse DRG neurons. These findings suggest that RJW-58 reverses oxaliplatin-induces CTSS production, activates the anti-inflammatory IL-10/STAT3 cascade, and reduces proinflammatory cytokines expression, which results in the alleviation of CIPN.

## Summary

This study highlights the novel role of CTSS in oxaliplatin-induced neurotoxicity, as evidenced by* in vitro* cell-line studies, *in vivo* OIPN mouse models, and clinical sample analysis. Blocking the enzymatic activity of lysosomal cathepsin S alleviates OIPN by regulating the STIM-dependent store-operated Ca^2+^ entry homeostasis. Furthermore, RJW-58, CTSS inhibitor, reverses oxaliplatin-induced CTSS production, activates the anti-inflammatory IL-10/STAT3 cascade, and reduces proinflammatory cytokines expression, which results in the alleviation of OIPN. RJW-58 might represent a potential therapeutic strategy for cancer patients with oxaliplatin-induced neuropathy.

## Conclusions

We demonstrated that CTSS plays a critical role in the development of oxaliplatin-induced neuropathy. Targeting CTSS may reveal a therapeutic strategy to protect neuron cells from oxaliplatin-induced damage. Furthermore, the serum level of CTSS may serve as a biomarker for the risk of oxaliplatin-induced peripheral neuropathies. A proof-of-concept large cohort prospective clinical study is ongoing.

## Supplementary Material

Supplementary figures.

## Abbreviations

ANOVA: analysis of variance
APCs: antigen-presenting cells
ATF3: activating transcription factor 3
BSA: bovine serum albumin
CTSS: cathepsin S
cDNA: complementary DNA
CRISPR/Cas9: clustered regularly interspaced short palindromic repeats/CRISPR-associated protein 9
CRC: colorectal cancer
CIPN: chemotherapy-induced peripheral neuropathy
ChIP: chromatin immunoprecipitation PCR
DMEM: Dulbecco's modified Eagle's medium
DNA: deoxyribonucleic acid
DRG: dorsal root ganglion
EDTA: ethylenediaminetetraacetic acid
ELISA: enzyme-linked immunosorbent assay
ER: endoplasmic reticulum
FACS: BD fluorescence-activated cell sorter
FBS: fetal bovine serum
FITC: fluorescein isothiocyanate
GAPDH: glyceraldehyde 3-phosphate dehydrogenase
GFP: green fluorescent protein
h: hour
HBSS: Hanks' balanced salt solution
HRP: horseradish peroxidase
HEPES: 4-(2-hydroxyethyl)-1-piperazine ethanesulfonic acid
IRF1: interferon response factor 1
IL-10: interleukin-10
IL-1β: interleukin-1β
IL-6: interleukin-6
IL-7: interleukin-7
i.p.: intraperitoneal(ly)
IENF: intraepidermal nerve fiber
JAKs: Janus tyrosine kinases
m: meter
M: molar
mRNA: messenger RNA
MCP1: monocyte chemoattractant protein 1
NF-κB: nuclear factor-κB
NP-40: Nonidet P-40
NCV: nerve conduction velocity
NET: nerve excitability test
OIPN: oxaliplatin-induced peripheral neuropathy
Olf-1: olfactory neuronal transcription factor 1
PAGE: polyacrylamide gel electrophoresis
PBS: phosphate-buffered saline
PCR: polymerase chain reaction
P300/CBP: p300 and CREB-binding protein
QST: quantitative sensory test
R: receptor
RNA: ribonucleic acid
RT: reverse transcription
ROS: reactive oxygen species
s: second
SDS: sodium dodecyl sulfate
SEM: standard error of the mean
siRNA: small interfering RNA
STAT3: signal transducer and activator of transcription 3
STIM1: stromal interaction molecule 1
SOCE: stored-operated Ca^2+^ entry
TNF-α: tumor necrosis factor-α
TNS: total neuropathy score
