# Supplementary material for: Targeting lysosomal cysteine protease cathepsin S reveals immunomodulatory therapeutic strategy for oxaliplatin-induced peripheral neuropathy
Source: Theranostics. 2021 Mar 4;11(10):4672–87. doi: 10.7150/thno.54793 (PMC7978314; doi:10.7150/thno.54793)
Supplement: Supplementary file 1 — Supplementary figures. [file thnov11p4672s1.pdf]

Supplementary Materials

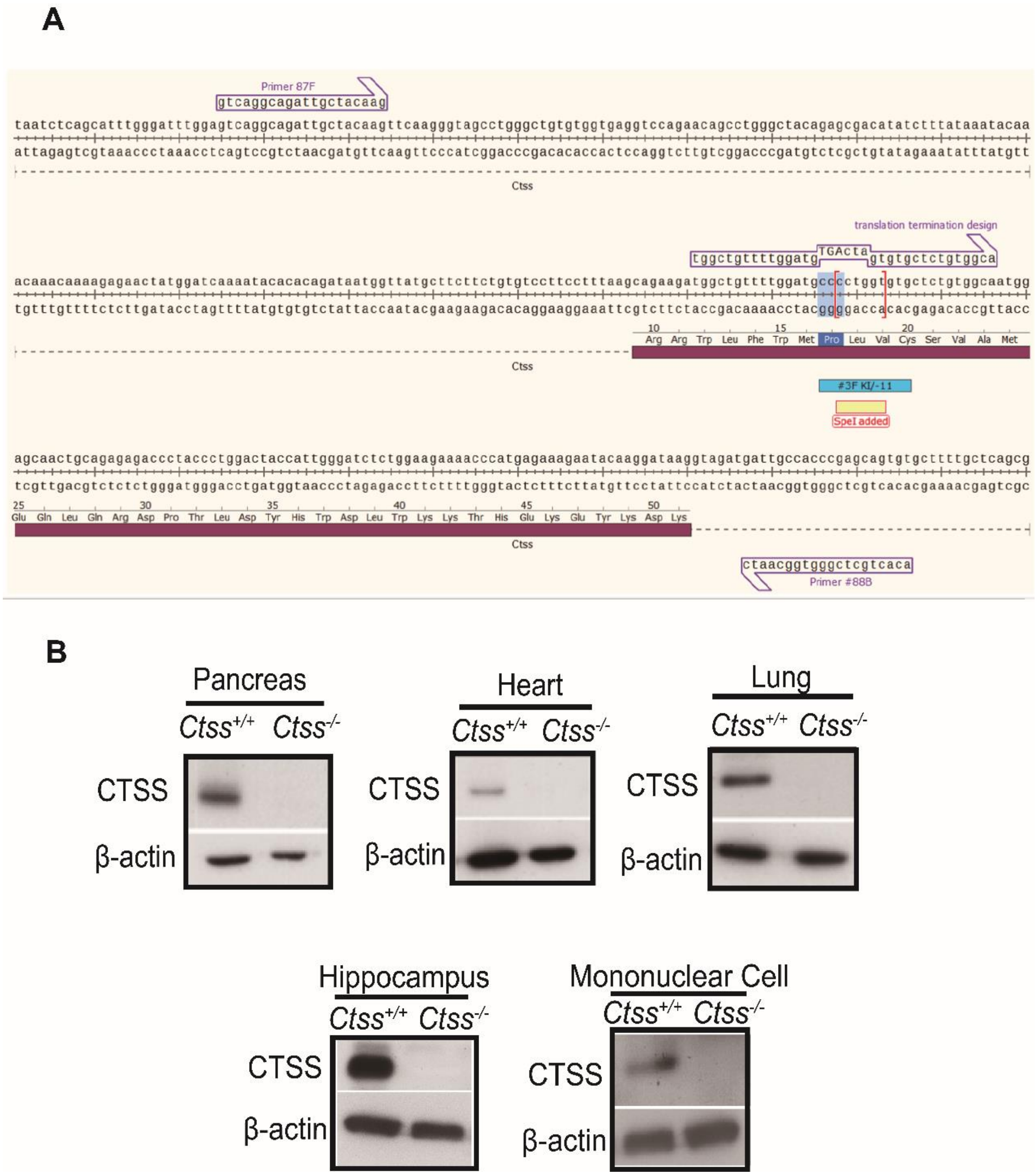

Figure S1. Establishment *Ctss* transgenic mouse model. A, Heterozygous *Ctss* mice were mated to obtain the F1 generation of *Ctss*$^{-/-}$ mice. The genotyping method is described in the Supplementary material and method section. B, Western blot analysis demonstrated that the expression of CTSS levels in different organs from *Ctss*$^{+/+}$ and F1 generation of *Ctss*$^{-/-}$ mouse.

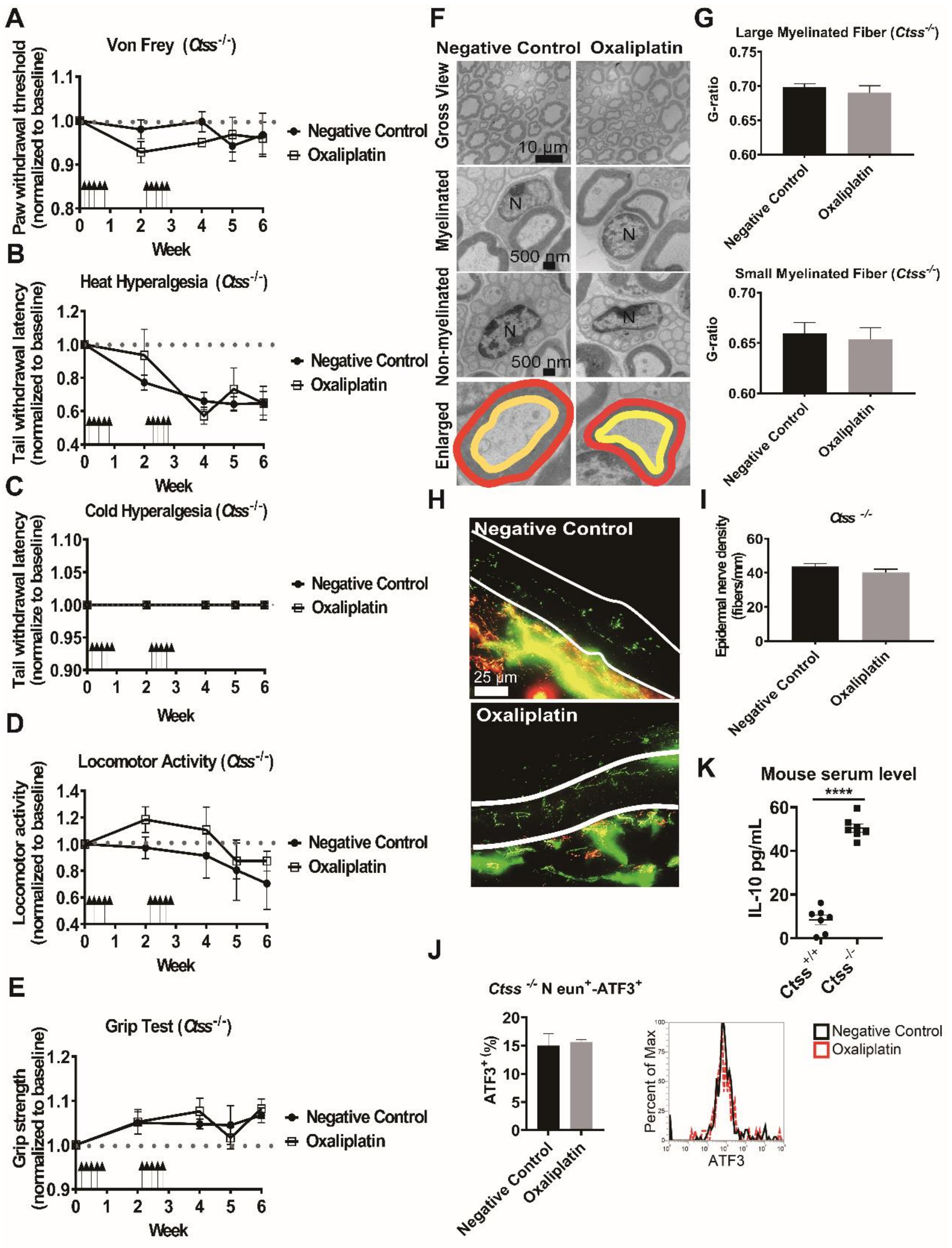

Figure S2. ***Ctss* deficiency protects mice from oxaliplatin-induced peripheral neuropathies.**

The basal levels of each behavioral assay were obtained before treatment. In the first week, the PBS control (100 μL) and oxaliplatin (3 mg/kg, 100 μL) were injected intraperitoneally five times per week for 2 weeks. Behavioral tests were performed weekly. A-E, *Ctss* deficiency reduced the oxaliplatin-induced mechanical allodynia, thermohypesthesia, and motor deficits. Right: quantification of the mechanical threshold, thermal sensitivity, and motor coordination at week 5 (n = 10 mice/group). Each value represents the mean ± SEM from at least 7 mice in each group. F-G, Transmission electron micrographs of the sciatic nerve from *Ctss*-knockout mice after oxaliplatin-induced axon loss at week 6 and demyelination. Scale bar, 10 μm (top), 500 nm (middle and bottom). Quantification of myelinated fiber density and the G-ratio in mouse sciatic nerves (n = 5 mice/group). H-I, Immunofluorescence images and quantification of intraepidermal nerve fibers suggest that the *Ctss* deficiency reduced the oxaliplatin-induced epidermal innervation loss at week 6 (n = 5 mice/group). Scale bar: 25 μm. J, Flow analysis of *Ctss*-knockout mice primary DRG cells at week 4 for $Neun^+$-$ATF3^+$ expression. K, The mouse serum IL-10 level between $Ctss^{+/+}$ and $Ctss^{-/-}$ mice. **** P < 0.0001, *** P < 0.001, ** P < 0.01, * P < 0.05 compared with baseline, each value represents the mean ± SEM, two-tailed Student's t-test.
